# Discovery of error-tolerant biclusters from noisy gene expression data

**DOI:** 10.1186/1471-2105-12-S12-S1

**Published:** 2011-11-24

**Authors:** Rohit Gupta, Navneet Rao, Vipin Kumar

**Affiliations:** 1 Department of Computer Science, University of Minnesota - Twin Cities, Minneapolis, MN 55455, USA

## Abstract

**Background:**

An important analysis performed on microarray gene-expression data is to discover biclusters, which denote groups of genes that are coherently expressed for a subset of conditions. Various biclustering algorithms have been proposed to find different types of biclusters from these real-valued gene-expression data sets. However, these algorithms suffer from several limitations such as inability to explicitly handle errors/noise in the data; difficulty in discovering small bicliusters due to their top-down approach; inability of some of the approaches to find overlapping biclusters, which is crucial as many genes participate in multiple biological processes. Association pattern mining also produce biclusters as their result and can naturally address some of these limitations. However, traditional association mining only finds exact biclusters, which limits its applicability in real-life data sets where the biclusters may be fragmented due to random noise/errors. Moreover, as they only work with binary or boolean attributes, their application on gene-expression data require transforming real-valued attributes to binary attributes, which often results in loss of information. Many past approaches have tried to address the issue of noise and handling real-valued attributes independently but there is no systematic approach that addresses both of these issues together.

**Results:**

In this paper, we first propose a novel error-tolerant biclustering model, ‘*ET-bicluster*’, and then propose a bottom-up heuristic-based mining algorithm to sequentially discover error-tolerant biclusters directly from real-valued gene-expression data. The efficacy of our proposed approach is illustrated by comparing it with a recent approach *RAP* in the context of two biological problems: discovery of functional modules and discovery of biomarkers. For the first problem, two real-valued *S.Cerevisiae* microarray gene-expression data sets are used to demonstrate that the biclusters obtained from *ET-bicluster* approach not only recover larger set of genes as compared to those obtained from *RAP* approach but also have higher functional coherence as evaluated using the GO-based functional enrichment analysis. The statistical significance of the discovered error-tolerant biclusters as estimated by using two randomization tests, reveal that they are indeed biologically meaningful and statistically significant. For the second problem of biomarker discovery, we used four real-valued *Breast Cancer* microarray gene-expression data sets and evaluate the biomarkers obtained using MSigDB gene sets.

**Conclusions:**

The results obtained for both the problems: functional module discovery and biomarkers discovery, clearly signifies the usefulness of the proposed *ET-bicluster* approach and illustrate the importance of explicitly incorporating noise/errors in discovering coherent groups of genes from gene-expression data.

## Background

Recent technical advancements in DNA microarray technologies have led to the availability of large-scale gene expression data. These data sets can be represented as a matrix *G* with genes as rows and different experimental conditions as columns, where (*G_ij_* denotes the expression value of gene *i* for an experimental condition *j.* An important research problem of gene-expression analysis is to discover submatrix patterns or biclusters in *G.* These biclusters are essentially subsets of genes that show coherent values across a subset of experimental conditions. However, coherence among the data values can be defined in various ways. For instance, Madeira et al [1] classify biclusters into the following four different categories based on the definition of coherence: (i) biclusters with constant values, (ii) biclusters with constant rows or columns, (iii) biclusters with coherent values, and (iv) biclusters with coherent evolutions. Many approaches [1-7] have been proposed to discover biclusters from gene-expression data. Different biclustering algorithms have been designed to discover different types of biclusters. For instance, coclustering [4] and SAMBA [5] find constant value biclusters, Cheng and Church (CC) [3] find constant row biclusters and OPSM [6] find coherent evolutions biclusters. Though there are differences in biclustering algorithms in terms of the type of bicluster they discover, there are some common issues with these algorithms in general. First critical issue with all of these biclustering algorithms is that they are oblivious to noise/errors in the data and require all values in the discovered bicluster to be coherent. This limits the discovery of valid biclusters that are fragmented due to random noise in the data. Second issue with at least some of the biclustering algorithms is their inability to find overlapping biclusters. For instance, coclustering is designed to only look for disjoint biclusters and Cheng and Church’s approach, which masks the identified bicluster with random values in each iteration, also finds it hard to discover overlapping biclusters. Third, most of the algorithms are top-down greedy schemes that start with all rows and columns, and then iteratively eliminate them to optimize the objective function. This generally results in large biclusters, which although are useful, do not provide information about the small biological functional classes. Finally, all the biclustering algorithms employ heuristics and are unable to search the space of all possible biclusters exhaustively.

Association pattern mining can naturally address some of the issues faced by biclustering algorithms i.e, finding overlapping biclusters and performing an exhaustive search. However, there are two major drawbacks of traditional association mining algorithms. First, these algorithms use a strict definition of support that requires every item (gene) in a pattern (bicluster) to occur in each supporting transaction (experimental condition). This limits the recovery of patterns from noisy real-life data sets as patterns are fragmented due to random noise and other errors in the data. Second, since traditional association mining was originally developed for market basket data, it only works with binary or boolean attributes. Hence it’s application to data sets with continuous or categorical attributes requires transforming them into binary attributes, which can be performed by using discretization [8-10], binarization [11-14] or by using rank-based transformation [15]. In each case, there is a loss of information and associations obtained does not reflect relationships among the original real-valued attributes, rather reflect relationships among the binned independent values [16].

Efforts have been made to independently address the two issues mentioned above and to the best of our knowledge, no prior work has addressed both the issues together. For example, various methods [17-26] have been proposed in the last decade to discover approximate frequent patterns (often called error-tolerant itemsets (ETIs)). These algorithms allow patterns in which a specified fraction of the items can be missing - see [27] for a comprehensive review of many of these algorithms. As the conventional support (i.e the number of transactions supporting the pattern) is not anti-monotonic for error-tolerant patterns, most of these algorithms resort to heuristics to discover these patterns. Moreover, all of these algorithms are developed only for binary data.

Another recent approach [28] addressed the second issue and extended association pattern mining for real-valued data. The extended framework is referred to as *RAP* (Range Support Pattern). A novel *range* and *range support* measures were proposed, which ensure that the values of the items constituting a meaningful pattern are coherent and occurs in a substantial fraction of transactions. This approach reduces the loss of information as incurred by discretization- and binarization-based approaches, as well as enables the exhaustive discovery of patterns. One of the major advantages of using an approach such as *RAP*, which adopts a very different pattern discovery algorithm as compared to more traditional biclustering algorithms such as *CC* or *ISA*, is the ability to find smaller or completely novel biclusters. Several examples shown in [28] illustrated that *RAP* can discover some biologically relevant smaller biclusters, which are either completely missed by biclustering approaches such as *CC* or *ISA*, or are found embedded in larger biclusters. In either case, they are not able to enrich the smaller functional classes as *RAP* biclusters do. Despite these advantages, *RAP* framework does not directly address the issue of noise and errors in the data.

As it has been independently shown that both issues, handling real-valued atributes and noise, are critical and affect the results of the mining process, it is important to address them together. In this paper, we propose a novel extension of association pattern mining to discover error-tolerant biclusters (or patterns) directly from real-valued gene-expression data. We refer to this approach as ‘*ET-bicluster*’ for error-tolerant bicluster. This is a challenging task because the conventional support measure is not anti-monotonic for the error-tolerant patterns and therefore limits the exhaustive search of all possible patterns. Moreover the set of values constituting the pattern in the real-valued data is different than the binary data case. Therefore, instead of using the traditional support measure, we used the *range* and *RangeSupport* measures as proposed in [28] to ensure the coherence of values and for computing the contribution from supporting transactions. *RangeSupport* is anti-monotonic for both dense and error-tolerant patterns, however, *range* is not anti-monotonic for error-tolerant patterns. Due to this, exhaustive search is not guaranteed, however it is important to note that the proposed *ET-bicluster* framework still, by design, finds more number of patterns (biclusters) than it’s counterpart *RAP.* Therefore using *range* as a heuristic measure, we describe a bottom-up pattern mining algorithm, which sequentially generates error-tolerant biclusters that satisfy the user-defined constraints, direcly from the real-valued data.

To demonstrate the efficacy of our proposed *ET-bicluster* approach, we compare it’s performance with *RAP* in the context of two biological problems: (a) functional module discovery, and (b) biomarker discovery. Since both *ET-bicluster* and *RAP* use same pattern mining framework, comparing them helps to quantify the impact of noise and errors in the data on the discovery of coherent groups of genes in an unbiased way.

For the first problem of functional module discovery, we used real-valued *S. cereυísíae* microarray gene-expression data sets and discovered biclusters using both *ET-bicluster* and *RAP* algorithm. To illustrate the importance of directly incorporating data noise/errors in biclusters, we compared the error-tolerant biclusters and *RAP* biclusters using gene ontology (GO) based biological processes annotation hierarchy [29] as the base biological knowledge. Specifically, for each {bicluster, GO term} pair, we computed a p-value using a hypergeometric distribution, which denotes the random probability of annotating this bicluster with the given GO term. For the second problem of biomarker discovery, we combined four real-valued case-control Breast Cancer gene-expression data sets, and discovered discriminative biclusters (or biomarkers) from the combined data set using both *ET-bicluster* and *RAP.* Again, to illustrate the importance of explicitly incorporating noise/errors in the data, we compared the biomarkers based on their enrichment scores computed using MSiGDB gene sets [30]. MSigDB gene sets are chosen as the base biological knowledge in this case because they include several manually annotated cancer gene sets. To further compare *ET-bicluster* and *RAP* algorithms, we also performed network/pathway analysis using IPA for an example biomarker obtained from each of the two algorithms. The results obtained for both the functional module discovery and biomarker discovery problem clearly demonstrate that error-tolerant biclusters are not only bigger than *RAP* biclusters but are also biologically meaningful. Using randomization tests, we further demonstrated that error-tolerant biclusters are indeed statistically significant and are neither obtained by random chance nor capture random structures in the data. Overall, the results presented for both the biological problems strongly suggest that our proposed *ET-bicluster* approach is a promising method for the analysis of real-valued gene-expression data sets.

## Contributions

• We proposed a novel association pattern mining based approach to discover error-tolerant biclusters from noisy real-valued gene-expression data.

• Our work highlights the importance of tolerating error(s) in the biclusters in order to capture the true underlying structure in the data. This is demonstrated using two case studies: functional module discovery and biomarker discovery. Using various real-valued gene expression data sets, we illustrated that our proposed algorithm *ET-bicluster* can discover additional and bigger biologically relevant biclusters as compared to *RAP.*

• We used two randomization techniques to compute the empirical p-value of all the discovered error-tolerant biclusters and demonstrated that they are statistically significant and it is highly unlikely to have obtained them by random chance.

**Organization:** The rest of the paper is organized as follows. In Section 2, we discuss our proposed algorithm *ET-bicluster.* Section 3 details the experimental methodology for evaluating the error-tolerant biclusters and their comparison with *RAP* biclusters, and the results obtained. We present a summary of the findings in section 4 followed by a discussion on limitations and future work in section 5.

## Experimental results and discussion

We implemented our proposed association pattern mining approach ‘*ET-bicluster*’ in C++. In this paper, we only compare our proposed approach with *RAP*, as *RAP* has already been shown to outperform biclustering approaches such as ISA and Cheng and Church, especially for finding small biclusters. Also, as mentioned in [28], transformation of data from real-valued attributes to binary attributes leads to loss of distinction between various types of biclusters (or patterns). Therefore, as the focus of this study is to discover constant row biclusters, binarization of real-valued gene-expression data is not meaningful. For this reason, we only show results on real-valued data sets. Further, in order to compare the performance of ‘*ET-bicluster*’ and *RAP* in discovering coherent groups of genes, we considered two biological problems: discovery of functional modules (finding coherent gene groups) and discovery of biomarkers (finding coherent gene groups that are discriminative of the two classes of patients: cases and controls).

**Selecting top biclusters:** As association mining based approach generally produces a large number of biclusters that often have substantial overlap with each other, this redundancy in biclusters may bias the evaluation. Hence, we used a commonly adopted selection methodology similar to the one proposed by [7] to select upto 500 top biclusters. However, because error-tolerant biclusters generally have a large set of supporting experimental conditions, even biclusters with high overlap in gene dimension may get selected in the top 500 biclusters. To avoid this situation, we computed the size of a bicluster by the number of genes (*|genes|*) in it, not by |*genes\* × |*conditions|* in it. Therefore, starting with the largest bicluster (only in terms of the number of genes in it), we greedily select upto 500 biclusters such that the overlap among any of the selected biclusters is not more than 25%. In case of a tie between the size of biclusters, bicluster with lower Mean Square Error (MSE) value [3] is selected. Please note that MSE of a bicluster is computed by discarding the error values in it, since *ET-bicluster* is meant to look for error-tolerant patterns.

### Case study 1 - discovery of functional modules

We used the following two real-valued *S. cereυísíae* microarray gene-expression data sets for the discovery of functional modules:

• Hughes et al’s data set [31]: This data set contains a compendium of expression profiles corresponding to 300 diverse mutations and chemical treatments in S. cerevisiae and was compiled to study the functions of yeast genes on a large scale. The overall dimensions of this data set are 6316 genes x 300 conditions, with values (*log*_10_ ratio of expression values observed for experimental condition and control condition) in the range [-2,2].

• Mega Yeast data set [32]: This data set contains 501 yeast microarray experiments, including stress responses, cell cycle, sporulation, etc. The overall dimensions of this data set are 6447 genes x 501 conditions, with values in the range [-12,12].

**Functional enrichment analysis:** Since the discovered biclusters represent groups of genes that are expected to co-express with each other, we evaluated all the biclusters discovered in terms of their functional coherence using the biological processes annotation hierarchy of Gene Ontology [29]. A p-value using a hypergeometric probability distribution is computed for each combination of bicluster and biological process GO term to determine if the discovered biclusters are statistically significant. The p-value computed for a pair of bicluster (denoted by *b*) and GO term (denoted by *t*) denotes the random probability of annotating a bicluster of size same as *b* with the same GO term *t.*

To compare error-tolerant biclusters and *RAP* biclusters in an unbiased fashion, we used the same 2652 biological processes GO terms (or classes), all of which contain at least 1 and at most 500 genes from *S.cerevisiae.* Furthermore, as only 4684 genes are annotated with either one or more of these 2652 classes, we restricted our analysis to a subset of data sets comprising of 4684 *genes* x 501 *conditions* and 4684 *genes* x 300 *conditions* for mega yeast and Hughes’s et al’s gene-expression data sets respectively.

#### Quantitative analysis of biclusters

Table 1 provides a general overview of all the biclusters obtained by *ET-bicluster* and *RAP* algorithm on mega yeast and Hughes et al’s real-valued gene-expression data sets using various parameter settings.

**Table 1:** This table shows various statistics of all the biclusters obtained using *RAP* and our proposed *ET-bicluster* algorithms from Mega Yeast and Hughes et al's microarray gene-expression data sets

Run ID	Parameter settings	# total biclusters	**# genes covered^1^**	**# top biclusters**	# genes covered^2^	**Size distribution^2^** *** #***** of genes:** ***# *** **of biclusters**	Time taken (seconds)
**Error-tolerant biclusters on Mega Yeast data set**							

*ET-bicluster_M_*_1_	*α* = 0.5, *ε*= 0 *for RS ε *[120 150), *ε* = 0.25 *for RS* ≥ 150	153,960	361	500	295	2:128, 3:235, 4:8, 5:76, 6:39, 7:7, 8:2, 9:1, 10:2, 11:1, 13:1	10,560

*ET-bicluster_M_*_2_	α = 0.3, *ε* = 0 for RS ∈ [60 90), *ε* = 0.25 *for RS* ≥ 90	271,101	792	500	233	3:203, 4:28, 5:177, 6:80, 7:5, 8:3, 9:3, 10:1	33,000

**RAP biclusters on Mega Yeast data set**							

*RAP_M1_*	α = 0.5, *RS* ≥ 120	33,330	361	500	247	2:68, 3:379, 4:33, 5:16, 6:4	642

*RAP_M_*_2_	*α* = 0.3, *RS* ≥ 60	94,806	792	500	241	3:384, 4:68, 5:43, 6:5	7,580

**Error-tolerant biclusters on Hughes et al's data set**							

*ET-bicluster_H_*_1_	*α* = 0.8, *ε* = 0 for RS ∈ [10 15), *ε* = 0.25 *for RS* ≥ 15	150,372	506	496	437	2:210, 3:187, 4:12, 5:66, 6:14, 7:3, 8:1, 10:1, 11:1, 13:1	8,360

*ET-bicluster_H_*_2_	*α* = 0.5, *ε* = 0 *for RS ∈* [6 10), *ε* = 0.25 *for RS* >10	234,761	1135	500	443	2:115, 3:258, 4:22, 5:69, 6:24, 7:6, 8:1, 9:2, 11:1, 13:1, 14:1	21,745

**RAP biclusters on Hughes et al's data set**							

*RAP_h_*_1_	*α* = 0.8, *RS* ≥ 10	56,009	506	495	438	2:212, 3:207, 4:25, 5:40, 6:5, 7:3, 8:2, 11:1	2,835

*RAP_h_*_2_	*α* = 0.5, *RS* ≥ 6	80,335	1135	500	405	2:96, 3:303, 4:18, 5:75, 6:2, 7:2, 8:3, 12:1	1,505

Statistics of biclusters obtained using '*ET-bicluster*' and '*RAP*' from Mega Yeast and Hughes et al's microarray gene-expression data sets. (^1 ^all biclusters, ^2^ top biclusters).

Parameter controlling error-tolerance (ε) was set to 0.25 in all the runs for *ET-bicluster.* It is important to note that number of error-tolerant biclusters is substantially larger than the number of *RAP* biclusters. Therefore, for a specific *range* (*α*) value and user-defined *Range Support* threshold, if *ET-bicluster* algorithm was not able to finish in a reasonable amount of time and memory with *α* = 0.25, we first obtain exact biclusters (no error-tolerance) by setting *α* to 0 and then increase the *RangeSupport* to obtain error-tolerant biclusters by setting *α* to 0.25. The final resulting set of biclusters is obtained by merging these exact and error-tolerant biclusters. Following are some of the general observations:

**Number of biclusters:** It can be clearly seen from Table 1 that introducing an error-tolerance of 25% substantially increased the total number of biclusters. For example, number of total error-tolerant biclusters obtained on mega yeast data is approximately 5-times (for *α* = 0.5) and 3-times (for *α* = 0.3) the number of *RAP* biclusters for corresponding *α* values. Similarly, for Hughes et al’s data set, number of error-tolerant biclusters is approximately 3-times the number of *RAP* biclusters for both the *α* values considered (*α* = 0.8 and *α* = 0.5).

**Size of biclusters:** Another important observation one can make from the results shown in Table 1 is that the size of error-tolerant biclusters is more than *RAP* biclusters. This is expected as *RAP* can only find exact biclusters (with no error-tolerance) and hence valid biclusters that are fragmented due to random noise and errors in the data, are either found as separate biclusters or completely missed. On the other hand, because *ET-bicluster* algorithm explicitly handles noise and errors in the data, it can potentially find larger biclusters by stitching together the fragmented parts or can even find new biclusters that were missed by *RAP.* This might have a significant impact on the functional enrichment analysis as *ET-bicluster* algorithm can potentially discover biclusters that have higher overlap with the considered GO biological processes classes. We discuss this further in the next section.

**Coverage of genes and relationships among them:** As can be noted from Table 1, the number of genes covered by *ET-bicluster* and *RAP* algorithm is same at least if we consider all biclusters. This is because the starting set of genes (‘singletons’) are same for both the algorithms. In fact, if the error-tolerance, *α* is 0.25 for example, then singletons, pairs (level-2 bicluster) and even triplets (level-3 bicluster) will be identical for *ET-bicluster* and *RAP.* However note that the number of level-4 biclusters generated by *ET-bicluster* is more than those generated by *RAP.* This is due to the fact that *ET-bicluster* algorithm, owing to its relaxed error-tolerance criterion, can generate more combinations of genes than *RAP.* Therefore in other words, even if the total genes covered by both the algorithms are same, *ET-bicluster* algorithm can find more relationships among them.

As mentioned above and shown in Table 1, since *ET-bicluster* algorithm, as compared *RAP*, can potentially find newer and larger biclusters and hence more relationships among genes, an important question to address is: whether these larger and new biclusters are biologically meaningful? One promising way to answer this question is through functional enrichment analysis and below we discuss these results.

#### Functional enrichment using GO biological processes

**Figure 1:**
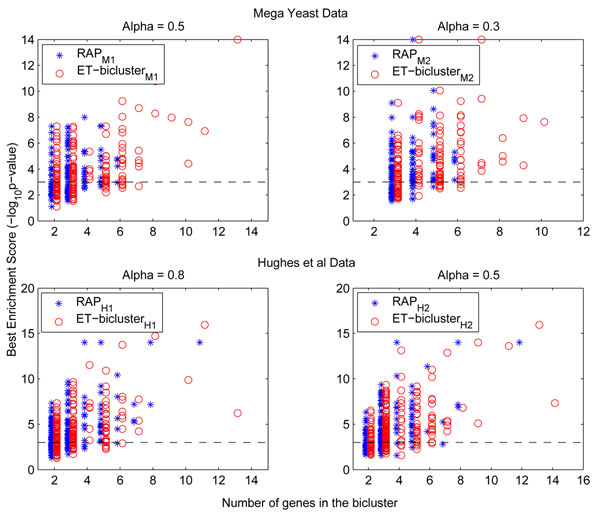
This figure shows the relationship between the size of biclusters and their enrichment scores as computed using GO biological processes for both Mega Yeast and Hughes et al’s data sets.

As mentioned earlier, a p-value for each of the (bicluster, GO term) pair is computed for the selected top 500 biclusters using the 2652 biological processes GO terms considered in this study. To demonstrate how well error-tolerant and *RAP* biclusters are enriched by GO terms, we show the distribution of *–log*_10_(*pvαlue*) and size of the biclusters. While Figures 1 (a) and (b) show this distribution for mega yeast data set corresponding to two *α* values of 0.5 and 0.3, Figures 1 (c) and (d) show this distribution for Hughes et al’s data set corresponding to *α* values of 0.8 and 0.5 considered in this study. It can be seen from these plots that *ET-bicluster* algorithm not only generates bigger biclusters (in terms of number of genes in them) as discussed before, but also these biclusters have high *–log*_10_(*pvalue*) (or low p-value), which means it is highly unlikely to have discovered these error-tolerant biclusters by random chance.

Consider mega yeast data for example, while *ET-bicluster* algorithm can discover biclusters of sizes as big as 13 (for *α* = 0.5) and 10 (for *α* = 0.3), *RAP* algorithm can only discover biclusters of maximum size 6. Moreover, enrichment scores of these larger error-tolerant biclusters (computed using the minimum p-value estimated for these biclusters for 2652 classes) are reasonably high. Therefore, even if the number of unique genes covered and number of enriched GO terms are comparable for *ET-bicluster* and *RAP* algorithm, the degree to which error-tolerant biclusters enrich the GO terms is certainly higher. In other words, *ET-bicluster* algorithm can find more relationships among the genes covered and as shown by functional enrichment analysis, these relationships indeed seem to be biologically relevant and not spurious.

Further, considering various p-value thresholds (from loose –5 × 10^–2^ to strict – 1 × 10^–5^), we collected two more statistics. First, the fraction of biclusters that are enriched by at least one GO term, and second, the fraction of GO terms that enriched at least one bicluster. To illustrate the efficacy of *ET-bicluster* in capturing the functional coherence among genes, and comparing it with *RAP*, the above two statistics are collected for all the runs shown in Table 1. For instance, if we compare these statistics for mega yeast data, while 83% of the top 500 error-tolerant biclusters (corresponding to Run ID *ET-bicluster_M_*_2_) were enriched, only 76% of the top 500 RAP biclusters (corresponding to Run ID *RAP_M_*_2_) were enriched by at least one GO term at a reasonable p-value threshold of 1 × 10^–3^, a gain of 7%. At even more strict p-value threshold of 1 × 10^–5^, the gain is 11%. Similarly, for Hughes et al’s data set, though the gain is not significant, biclusters obtained from *ET-bicluster* still outperform those obtained by *RAP* in terms of the fraction of biclusters enriched. As far as the second statistics is concerned i.e. the number of GO terms that enriched at least one bicluster, performance of *ET-bicluster* and *RAP* is comparable, however, as shown in *–log*_10_(*pvalue*) vs. *size* distribution plots, enrichment scores for error-tolerant biclusters are generally higher than *RAP* biclusters.

#### Statistical significance of error-tolerant biclusters using randomization tests

Motivated by the discussion of randomizaton tests and their importance in validating the results from any data mining approach [33], we further estimate the statistical significance of the error-tolerant biclusters using a data centric randomization approach. More specifically, an empirical p-value is computed for all the error-tolerant biclusters using the two randomization tests.

In the first randomization test, conserving the size of the top 500 error-tolerant biclusters, we generated 1000 random sets of 500 biclusters each and evaluated them by the same functional enrichment analysis using GO biological processes. So effectively, for each actual error-tolerant bicluster, we generated 1000 random biclusters of the same size (in terms of number of genes). The empirical p-value for each actual error-tolerant bicluster is then computed as the fraction of random biclusters (out of total 1000) whose enrichment score (*–log*_10_(*pvalue*)) exceeds the enrichment score of the actual error-tolerant bicluster. For instance, if for a error-tolerant bicluster, only 1 out of 1000 random biclusters has higher enrichment score than it’s actual value, empirical p-value of this error-tolerant bicluster is given as ‘1 *in* 1000’ or 10^–3^.

**Figure 2:**
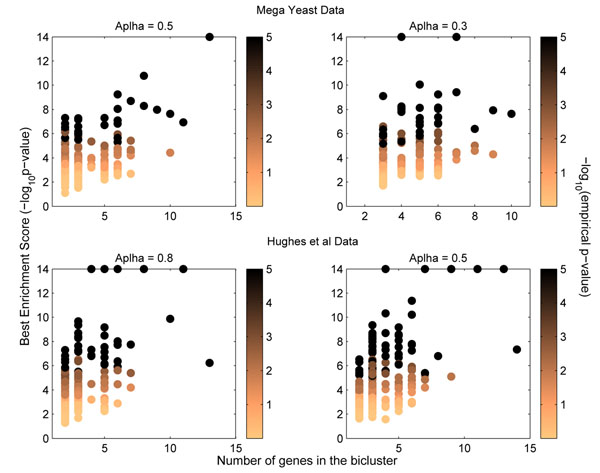
This figure shows the biological and empirical p-values (using 1000 random runs) of the biclusters obtained using our proposed *ET-bicluster* algorithm. This figure is best viewed in color.

Figure 2 shows the (*–log*_10_(*empiricαl p-values*)) for all the error-tolerant biclusters that were shown in Figure 1. To plot these values at the same scale, an empirical p-value of ‘0 *in* 1000’ is set to 10^–5^ to ensure that they stand out from the rest. Therefore, all the biclusters showing (*–log*_10_(*empirical p-values*)) as 5 in Figure 2 correspond to empirical p-value of ‘0 *in* 1000’. It can be clearly seen from Figure 2 that error-tolerant biclusters that were assigned high enrichment scores from the GO-based evaluation also have high (*–log*_10_(*empirical p-values*))*.* This means higher the enrichment score of a bicluster, less likely it is to obtain this by random chance, which further illustrates that the bigger error-tolerant biclusters discovered by only *ET-bicluster* algorithm but not by *RAP* algorithm are indeed statistically significant.

We also showed in Table 2, the summary statistics of the evaluation results on 1000 randomly generated sets of biclusters. More specifically, for a given p-value threshold, we first compute for each of the 1000 random runs, the fraction of biclusters that have a p-value better than the given threshold and then we report how many times it exceeds the same fraction computed for the actual set of biclusters. It can be clearly seen from the Table that specially for a stricter p-value threshold, none of the randomly generated biclusters are better than the actual biclusters. For instance, while 83% of the actual 500 biclusters on mega yeast data (‘Run ID: *ET-bicluster _M2_*’) had *–log*_10_(*pvalue*) higher than 3, this percentage for 1000 random runs was substantially lower with mean of around 36% and a maximum of only 42%. The results were very similar for Hughes et al’s data set. Both these set of results further confirms the statistical significance of biclusters obtained from *ET-bicluster* algorithm.

**Table 2:** This table shows the statistical significance of biclusters obtained from our proposed *ET-bicluster* algorithm

Run ID	*#* of random runs out of 1000 in which fraction of biclusters enriched exceeds the fraction for the true run				
	pval ≤ 0.05	pval ≤ 0.01	pval ≤ 0.005	pval ≤ 0.001	pval ≤ 0.00001

*ET-bicluster_M_*_1_	660	33	0	0	0
*ET-biduster_M_*_2_	660	76	4	0	0
*ET-bicluster_H_*_1_	797	0	0	0	0
*ET-bicluster_H_*_2_	886	0	0	0	0

Statistical significance of biclusters obtained from *ET-bicluster*.

In the second randomization test, we randomized the data itself by shuffling the data values among the conditions for each gene. By doing this, we conserved the distribution of each gene profile but broke the correlation among them. We ran our proposed *ET-bicluster* algorithm on randomized mega yeast data set for example, and obtained only 42 biclusters, all of which were pairs. In contrast, application of *ET-bicluster* algorithm on actual non-randomized mega yeast data generated many more biclusters and of size as big as 10.

Both of the above randomization tests suggest that the error-tolerant biclusters obtained from the real-valued gene-expression data sets were indeed biologically meaningful and are neither obtained by random chance nor capture random structures in the data.

### Case study 2 - discovery of biomarkers

We used four real-valued *Breast Cancer* gene-expression data sets, all of which were taken from Affymetrix platform HGU133A and normalized using RMA-normalization approach. Please note that these gene-expression data sets are different than those considered for functional module discovery problem, in the sense that experimental conditions are replaced by two groups of patients. All the four breast cancer data sets were downloaded from GEO website: Desmedt (GSE7390), Loi (GSE6532), Miller (GSE3494) and Pawitan (GSE1456). The patients in the four data sets are classified as cases and controls based on their metastasis state. The patients who developed metastasis within 5 years of prognosis were considered as metastasis cases. The patients who were free of metastasis longer than 8 years of survival and follow-up time were considered as controls. The case-control ratio for Desmedt, Loi, Miller and Pawitan data set was 35:136, 51:112, 37:150 and 35:35 respectively. To increase the samle size, we combined these four data sets and used it for the discovery of biomarkers. This combined data set comprises of 8,920 genes and a case-control ratio of 158:433.

We discovered biclusters on combined Breast Cancer gene-expression data set using *ET-bicluster* with parameters, *α* = 0.5, *RS* = 80, and *α* = 0.25.

**Selecting disriminative biclusters:** First we select top biclusters using the approach defined earlier and then amongst the top biclusters, only those are selected as biomarkers that are discriminative of the two groups of patients, cases and controls. To measure the discriminative power, we used two measures, odds ratio and p-value. While odds ratio quantifies how different are cases and controls for a specific bicluster, p-value quantifies the significance of the difference reflected by odds ratio. Only those biclusters are selected that have a p-value of less than 0.05 and odds ratio of more than 2.0 (biclusters more represented in cases) or less than 0.5 (biclusters more represented in controls).

**Functional enrichment analysis:** We evaluated all the identified biomarkers in terms of their enrichment scores using the MSigDB gene sets [30]. A p-value using a hypergeometric probability distribution, which denotes the random probability of annotating a biomarker with the gene set considered, is computed for all pair combinations of biomarkers and 5452 gene sets from MSigDB database. Enrichment score of each biomarker is then computed as *–log*_10_(*p-value_min_*) and used as a metric to compare the biomarkers obtained using *ET-bicluster* and *RAP.*

### Enrichment analysis using MSigDB gene sets

**Figure 3:**
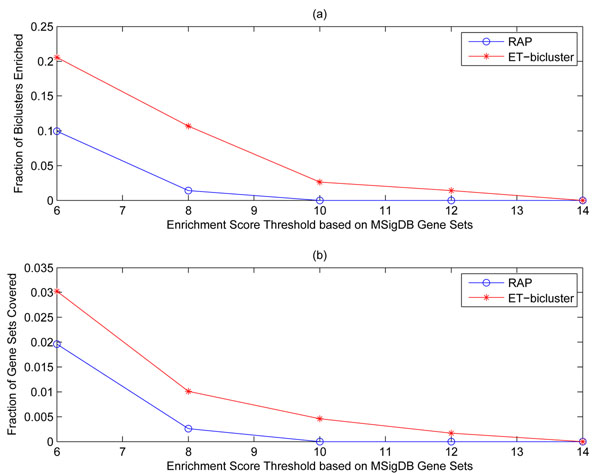
(a) This figure shows the fraction of biomarkers enriched by at least one MSigDB gene set. (b) This figure shows the fraction of MSigDB gene sets enriched by at least one biomarker.

Considering various p-value thresholds (from 10^–6^ to 10^–14^), Figure 3 shows two statistics: (a) fraction of biomarkers enriched by at least one gene set, and (b) fraction of gene sets that enriched at least one biomarker. These two statistics are collected both for biomarkers obtained from *ET-bicluster* and *RAP* algorithm at various p-value thresholds. Note that since the main goal of this analysis is to just compare the biomarkers obtained from *RAP* and *ET — bicluster* algorithms, p-values are not corrected for multiple hypothesis testing. As mentioned earlier, biomarkers obtained by *ET-bicluster* are not only bigger than those obtained by *RAP*, as illustrated in Figure 3(a), even a higher fraction of them is enriched by at least one gene set. Consider for instance, a strict p-value threshold of 10^–8^ (corresponding to *–log*_10_(*p-value*) of 8 as shown on the x-axis), while 10.5% of the error-tolerant biomarkers are enriched, only 1.5% of the RAP biomarkers are enriched.

Now refer to Figure 3(b), gene sets covered by *ET-bicluster* biomarkers are more than those covered by *RAP* biomarkers. The fraction of gene sets covered by biomarkers obtained from both the algorithms seems very low but this is expected because first a large number of gene sets are considered for the analysis and second, these biomarkers are only reflective of breast cancer metastasis. An important point to note however is that even a small change in fraction of gene sets covered would mean covering substantially large number of gene sets. For instance, consider a p-value threshold of 10^–6^ (corresponding to *–log*_10_(*p-value*) of 6 as shown on the x-axis), *ET-bicluster* and *RAP* biomarkers cover 3.03% (165 gene sets) and 1.96% (107 gene sets) respectively. These numbers for a even stricter p-value threshold of 10^–8^ are 1.01% (55 gene sets) 0.26% (14 gene sets) respectively.

**Figure 4:**
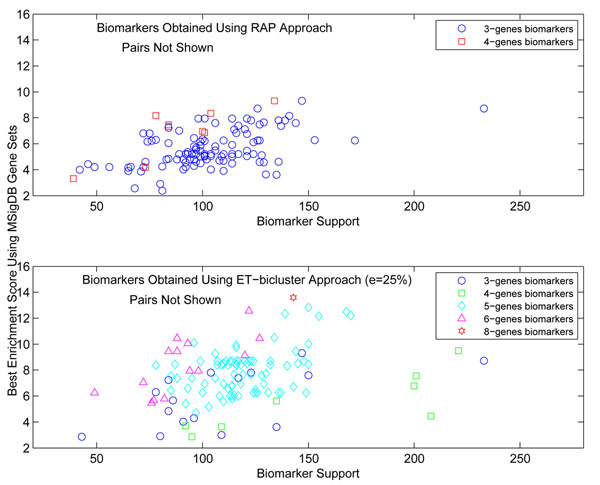
This figure shows the relationship among enrichment score computed using MSigDB gene sets, support (number of samples supporting the biomarker) and size (number of genes) of biomarkers obtained using *RAP* and *ET-bicluster* algorithms.

After observing these global statistics for biomarkers obtained using *RAP* and *ET-bicluster* algorithms, we further dig deeper to analyze the enrichment score, support (number of samples supporting the biomarker) and size (number of genes in biomarker) of each biomarker obtained using these two algorithms. Figure 4 shows the relationship among the above variables for biomarkers obtained using *RAP* (top plot) and *ET-bicluster* (bottom plot) algorithms. It is quite clear from the figure that biomarkers obtained using *ET-bicluster* algorithm are bigger in size as compared to those obtained using *RAP* algorithm. This, as stated before, is not unexpected but an important observation is that biomarkers obtained using *ET-bicluster* algorithm are supported by more number of samples. Although due to patient heterogeneity and several other factors, it is understandable that biomarkers may not have very high support, but nevertheless higher support of a biomarker generally translates to its better clinical utility. Therefore it is quite encouraging to observe from Figure 4 that biomarkers obtained from *ET-bicluster* algorithm are not just bigger compared to *RAP* biomarkers but a higher fraction of them have higher support as well as higher enrichment score.

It is clear from the above analysis that the biomarkers obtained from *ET-bicluster* algorithm are indeed biologically meaningful and since *RAP* algorithm does not explicitly handle noise in the data, it either completely miss some of these biologically relevant biomarkers or find fragmented parts of these, which eventually affect their enrichment score.

#### Biological relevance - example

**Figure 5:**
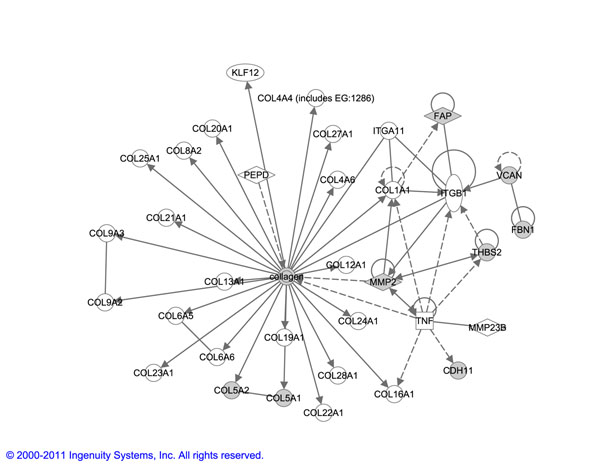
This figure shows the top network enriched based on an example biomarker (8 genes) obtained using our proposed *ET-bicluster* algorithm.

We also observed the network based enrichment for an example biomarker obtained by each of the algorithms, *ET-bicluster* and *RAP.* Data were analyzed through the use of Ingenuity Pathways Analysis (Ingenuity Systems, www.ingenuity.com). We chose the biggest biomarker obtained by *ET-bicluster* algorithm, which comprises of 8 genes: *CDH*11 *COL*5*A*1 *COL*5*A*2 *FAP FBN*1 *MMP*2 *THBS*2 *VCAN.* We also selected the corresponding biggest biomarker obtained by *RAP* algorithm, which comprises of 4 genes: *COL*5*A*1 *COL*5*A*2 *FAP VCAN.* As can be seen, *RAP* biomarker is a subset of *ET-bicluster* biomarker. As shown in Figure 5, all the 8 genes in *ET-bicluster* biomarker are assembled into a network containing the collagen family of genes and *Intergin — β*1 (ITGB1) signaling, indicating that an interaction between the *Inter gin — β*1 signaling pathway and regulation of collagen genes might be important for breast cancer metastasis. Collagen is a core component of the extracellular matrix (ECM).

**Figure 6:**
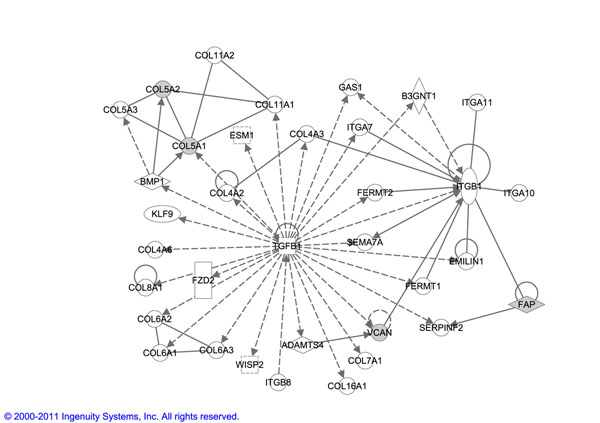
This figure shows the top network enriched based on an example biomarker (corresponding to the one obtained using ET-bicluster algorithm and considered in Figure 5) obtained using *RAP* algorithm.

During metastasis, tumor cells can interact with the ECM through adhesion molecules such as integrins. In fact, *Integrin — β*1 expression has previously been significantly associated with lymph node metastasis in non-small cell lung cancer patients [34]. In comparison, the top network obtained for *RAP* biomarker (shown in Figure 6), which is a complete subset of *ET-bicluster* biomarker, also contains quite a few collagen family of genes and ITGB1 signaling components. However, in order to connect these two components, *TGF — β*1 (TGFB1) is also included in the network even though none of the genes surrounding TGFB1 are enriched for this biomarker. This requirement for TGFB1 to be included in the network is excluded in pattern *ET-bicluster* biomarker since the *MMP*2 gene, which is a known breast cancer biomarker, acts as a nice connector between the collagen family of genes and ITGB1 signaling.

Thus the network obtained by the bigger *ET-bicluster* biomarker is better connected and therefore has a higher network score as computed using IPA than that obtained from *RAP* biomarker. In fact, all the 4 additional genes in *ET-bicluster* biomarker i.e. *MMP*2, *CDH*11, *THBS*2 and *FBN*1 are previously shown to be well-characterized cancer biomarkers (as identified in IPA), increasing our confidence that the bigger *ET-bicluster* biomarker is indeed a true biomarker.

## Conclusions

We proposed a novel error-tolerant biclustering model and presented an heuristic-based algorithm ‘*ET-bicluster*’ to sequentially generate error-tolerant biclusters from real-valued gene-expression data in a bottom-up fashion.

We presented two biological case studies, functional module discovery and biomarker discovery, to demonstrate the importance of incorporating noise and errors in the data for discovering coherent groups of genes. In both the case studies, we found that the biclusters discovered using our proposed *ET-bicluster* algorithm are not only bigger than those obtained by *RAP* algorithm, they were also assigned a higher functional enrichment score using the biological processes GO terms (functional module discovery case study) and MSigDB gene sets (biomarker discovery case study). These results suggest that the discovered error-tolerant biclusters, not only capture the functional coherence among the genes, it is unlikely to have obtained them by random chance. We further demonstrated using two randomization tests that the statistical significance of error-tolerant biclusters is high. The results from both randomization tests (one randomly selects the biclusters and other randomizes the input data itself) suggest the robustness of our proposed approach and clearly illustrate that discovered biclusters were indeed biologically and statistically meaningful and neither obtained by random chance nor capturing any random structure in the data.

The work presented in this study can be extended in various ways. Below we discuss some of the limitations of the *ET-bicluster* algorithm and possible ideas to address them.

• Since the *range* criterion that is used to check the coherence of expression values is not anti-monotonic, the proposed *ET-bicluster* approach does not exhaustively search for all error-tolerant biclusters. Therefore, a promising idea is to define a new anti-monotonic measure that measures the coherence among the expression values and enable exhaustive search for error-tolerant biclusters.

• The current implementation of *ET-bicluster* algorithm only impose error-tolerance constraints in the bicluster row. This means that it is possible for a gene in a discovered bicluster to have all error values. To avoid this situation, one can use additional column constraint and find a subset of supporting transactions for which each column in the pattern has no more than some user-defined fraction of errors. For binary data case, this kind of additional column constraint has been used in [20], however, a heuristic-based approach is used to check the column constraint. One of the promising directions is to develop a pattern mining algorithm that imposes both the row and column error-tolerance constraints, and exhaustively search for all the error-tolerant biclusters.

We only presented comparison of *ET-bicluster* and *RAP* since comparison with other biclustering approaches such as *CC* and *ISA* is not well suited for quantifying the affect of noise/errors. Moreover *CC* and *ISA* approaches generally finds larger biclusters and follow a different approach based on optimizing an objective function. Nevertheless, it will still be interesting in future to compare *ET-bicluster* with *CC* and *ISA* for potential complementarity among them.

It is also important to note that gene-expression data provides useful but limited view of the genome and therefore biclusters obtained from gene-expression data alone may not elucidate the complete underlying biological mechanism. Therefore to further illustrate the utility of *ET-bicluster* algorithm, another promising research direction is to integrate multiple biological data sources. For example, protein-protein interaction data can be used as a prior knowledge to guide the discovery of biclusters from the gene-expression data. The biclusters identified in this way are potentially more reliable and biologically plausible than those obtained from individual data sources. We are currently developing error-tolerant pattern mining based approaches for integrated analysis of gene-expression and protein-protein interaction data. Our initial efforts to combine these two sets of data sets for discovering sub-network based biomarkers has been shown in [35], however, these approaches are primitive at this stage and further work is needed in this area.

## Methods

### Error-tolerant bicluster model for real-valued data

As shown in [1], there can be different types of biclusters one can define on a real-valued data based on different measures of coherence among data values. In this paper, we focus on constant row/column biclusters, as they are well suited for the *ET-bicluster* framework and also considered as one of the promising ways to capture functional coherence from the microarray data sets [3]. However, discovering error-tolerant biclusters directly from real-valued data is a challenging task as several issues arise either due to handling of real-valued attributes or due to relaxing the bicluster requirements to incorporate noise/errors in the data. Specifically, following three issues need to be discussed before we present the algorithm.

**Figure 7:**
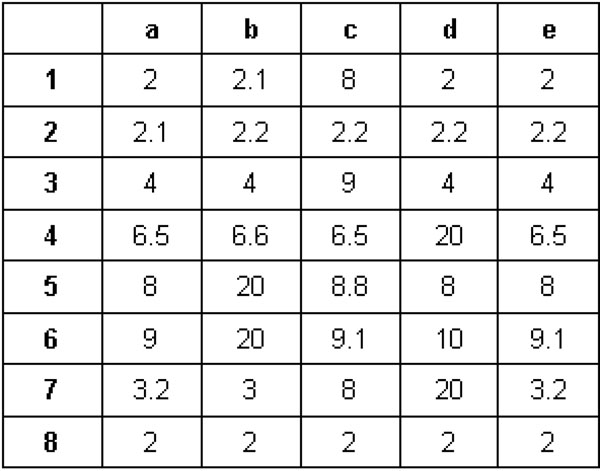
A sample matrix showing an example of error-tolerant bicluster.

(a) **Bicluster composition:** Unlike the case of binary data where collection of 1s was defined as a bicluster, in the case of real-valued data, similar values across a set of rows constitute a bicluster. These values can be any values in the set ℝ and athough similar across rows, they can be different for different rows. The errors in the biclusters defined on real-valued attributes are introduced in a way similar to the binary case. However, like binary case in which all non-error entries are same (1s), in real-valued case, imposing such a requirement would be very harsh. Therefore, a measure is needed to check the coherence among the gene-expression values. For this purpose, we use the *range* measure, which checks for each transaction if the relative range of the gene-expression values in a bicluster, given as (*max_val_ –min_val_*)*/min_val_*, is within a pre-specified threshold *α.* Furthermore, the contribution of each supporting transaction is measured as the minimum of the values taken by any of the genes in the bicluster in that transaction. Overall, to measure the strength of the bicluster, we use the *RangeSupport* (*RS*) measure [28], which sums up the contribution of each supporting transaction. This is similar to the *support* measure that is generally used in association pattern mining for binary data, however unlike binary case, each supporting transaction may not contribute equally for *RangeSupport* of a bicluster in real-valued data. The *range* and *RangeSupport* measures in combination capture the requirement that expression values of the genes in a bicluster are coherent for several transactions, and hence can be used to mine interesting biclusters from the real-valued data. Note that although both measures *range* and *RangeSupport* are anti-monotonic for exact biclusters, *range* is not anti-monotonic for error-tolerant biclusters. Due to this reason, *ET-bicluster* does not exhaustively find all error-tolerant biclusters, but it is noteworthy that it still subsume all biclusters found by *RAP* and can even find biclusters that are fragmented due to noise/errors in the data. One the other hand, as *RAP* is oblivious to errors/noise in the data, it either completely miss these fragmented but valid biclusters or find them as separate parts.

(b) **Positive/negative values:** Unlike binary data, real-valued microarray data has both positive and negative values. In this case, it is important to consider the sign of the value to discover meaningful biclusters. Similar to [28], we address this problem by enforcing that a transaction can only be termed as the supporting transaction of a bicluster if for this transaction, the expression values of all the genes in the bicluster are of the same sign. This also help make biological interpretability easier as the sign enforcement would entail finding only those biclusters in which all the genes are either up-regulated or down-regulated for a given experimental condition. However note that the same genes can be up-regulated for one experimental condition and down-regulated for another.

(c) **Error/non-error values:** In binary case, 1 is always a non-error value and 0 an error value. This notion is no more valid for the real-valued data case. For example, consider an error-tolerant bicluster shown in Figure 7 with 5 genes (a, b, c, d, e) and 8 experimental conditions (1 … 8). For the 1st condition, 8 is an error value, for the 3rd condition 9 is an error value, and for the 5th condition, 20 is an error value. Similarly, non-error values can change for each transaction. Thus, it becomes important to keep track of error and non-error values while mining for biclusters in the real-valued data.

Now, with the understanding of specific challenges and potential ways to address them, we now give the formal definition of error-tolerant biclusters for a real-valued data.

### Definition of error-tolerant biclusters

Intuitively, a bicluster *B* is said to be an error-tolerant bicluster if the following two general conditions are satisfied:

• *RangeSupport* of bicluster *B* should be more than the user-defined threshold, *RS.*

• All supporting transactions of bicluster *B* should have mostly non-error values i.e. values should be generally coherent (governed by a user-defined parameter *ε* for maximum number of permissible errors).

*Definition* 1. Let *D* be a real-valued gene-expression data, *RS* be the *RangeSupport* threshold, *E* be a function that takes a set of real values as input and returns the number of errors in them using *range* criteria, and let error threshold be ε ∈ (0,1]. A bicluster *B* (with genes *G*) is an error-tolerant bicluster *ET-bicluster*(*ε*) in the real-valued attribute domain, if there exists a set of transactions *T* ∈ *D* such that the following two conditions hold:

*Range Support* (*B*) ≥ *RS* (1)

∀*t* ∈ *T*,*E* (*D_t_*_,_*_G_*) ≤ *ε • |G|* (2)

Thus according to the definition, fraction of errors in each supporting transaction of the bicluster should not exceed *ε*.

### Algorithm to discover error-tolerant biclusters from real-valued data

Starting with singletons, the *ET-bicluster* algorithm sequentially generates (k+l)-level biclusters from k-level biclusters. At k = 1, genes that satisfy the *RangeSupport* (computed as the summation of absolute values for all transactions) criterion are valid singletons. Generally speaking, any (k+1)-level bicluster is a valid bicluster if it satisfies the *RangeSupport* criterion and each supporting transaction of the bicluster has at most *ε* fraction of errors.

*ET-bicluster* algorithm generates (k+1)-level biclusters from k-level biclusters by one of the two steps: error extension or non-error extension. Specifically, if ⌊ (*k* + 1) * *ε*⌋ = ⌊*k* * *ε*⌋, it’s a non-error extension step (no more errors values are permitted) or else it will be a error-extension step (one additional error value is permitted). We used two lemmas proved in [20] to efficiently perform these extension steps. In non-error extension step, for each (k+l)-level bicluster, *range* criteria is only checked for the intersection of supporting transactions of all its k-level biclusters. On the other hand, in the error-extension step, *range* criteria is checked for the union of supporting transactions of all its k-level biclusters.

Checking the range criterion to ensure the coherence of values depends on the number of permissible errors at a particular bicluster-level (*k* • *ε*). For instance, if the permissible number of errors is 1, then *range* criterion for a given transaction is computed as follows. First, for each transaction, all the expression values in a bicluster are sorted and then the range criterion is checked in usual manner by either discarding the minimum value or the maximum value. If the *range* criterion is satisfied in any of the two cases, transaction is classified as the supporting transaction for that bicluster. If for instance, number of permissible errors are 2 at any bicluster-level, we check the *range* criterion for three cases: discarding the 2 minimum values; discarding the 2 maximum values; or discarding 1 minimum value and 1 maximum value.

Again, if any of the case satisfies the *range* criterion, transaction is classified as a supporting transaction. Similarly, we exhaustively make all cases when number of permissible errors are more than 2. However, note that with *ε*= 0.25 (value considered in this paper) and bicluster size in terms of number of genes even as big as 12, we only need to make these cases for 3 permissible errors.

## An example

Considering a sample real-valued data with 5 genes (a, b, c, d, and e) and 8 experimental conditions (1 through 8) as shown in Figure 7, below we demonstrate the steps of *ET-bicluster* algorithm. Input parameters: Range Support threshold = 5; *α* = 0.5; *ε* = 0.25.

**Step 1: ***k* = 1. As range support for each gene is greater than 5, all the genes are returned as valid singletons.

**Step 2: ***k* = 2. Since ⌊k * e⌋ = ⌊*k –* l⌋ * *ε*, this is a non-error extension step. Consider for example bicluster *ab*, for *α* = 0.5, it’s supporting transactions are {1,2,3,4,7,8}. To illustrate, while transaction 1 satisfies the range criteria (i.e. 2.1 — 2 < 0.5 * 2) and hence is valid, transaction 5 is not valid since 20 — 8 > 0.5 * 8. Now, *RangeSupport* of bicluster *ab* is given as the sum of the contributions from each supporting transaction i.e. *RS*(*ab*) = 2 + 2.1 + 4 + 6.5 + 3 + 2 = 19.6. Since, *RS*(*ab*) > 5, *ab* is a valid bicluster. Similarly, biclusters *ac*, *ad*, *ae*, *bc*, *bd*, *be*, *cd*, *ce*, *de* are all valid biclusters.

**Step 3: ***k* = 3. Again since ⌊*k* * ε⌋ = ⌊*k –* l⌋ * *ε*, this is a non-error extension step. Consider for example, bicluster *abc*, *range* criterion is checked for intersection of supporting transactions of biclusters *ab*, *bc* and *ac* and hence supporting transactions are identified as {2,4,8}. Now, since *RS*(*abc*) = 10.6, which is greater than the thereshold 5, *abc* is a valid bicluster. Similarly, *abd*, *abe*, *bee*, *bde* and *cde* are all valid biclusters.

**Step 4: ***k* = 4. In this case, since ⌊k * ε⌋ ≠ ⌊*k –* 1⌋ * *ε*, this is an error extension step. The number of permissible errors at this level is k * ε_r_ = 4 * 0.25 = 1. Consider for example, bicluster *abcd*, *range* criterion is checked for the union of supporting transactions of all its level-3 biclusters subsets. Hence, we get {1,2,3,4,5,6,8} as the set of supporting transactions. For illustration, take an example of transaction 1. As only one error value is permitted, range criterion is checked as follows:

(((2*^nd^max* – *min*)*/min*) = (2.1 – 2)/2 = 0.05 < α(0.5)). Therefore, this is a supporting transaction. On the other hand, transaction 7, even after discarding one error value does not satisfy the range criterion for bicluster *abcd.* Also *RS*(*abcd*) = 33.6, hence *abcd* is a valid bicluster. Similarly, *abce* is also a valid bicluster.

**Step 5: ***k* = 5. Since, ⌊k * e⌋ = ⌊*k –* l⌋ * *ε*, this is a non-error extension step. A bicluster *abcde* will be generated with set of supporting transactions as {1,2,3,4,5,6,8}. Now since *RS*(*abcde*) = 33.6, abcde is a valid bicluster.

It is important to note that since *RAP* does not explicitly handle errors/noise in the data, it cannot discover the bicluster *abcde*, which is fragmented due to errors.

## Authors' contributions

RG and VK conceived and designed the study. RG, NR and VK developed the proposed approach and the evaluation methodologies. RG and NR prepared the implementation and experimental results. All the authors participated in the preparation of the manuscript and approved the final version.

## Competing interests

The authors declare that they have no competing interests.
